# Draft Genome of *Pseudomonas stutzeri* Strain NF13, a Nitrogen Fixer Isolated from the Galapagos Rift Hydrothermal Vent

**DOI:** 10.1128/genomeA.00113-13

**Published:** 2013-03-21

**Authors:** Arantxa Peña, Antonio Busquets, Margarita Gomila, Joan Mayol, Rafael Bosch, Balbina Nogales, Elena García-Valdés, Antonio Bennasar, Jorge Lalucat

**Affiliations:** a Microbiologia, Departamento de Biologia, Universitat de les Illes Balears, Campus UIB, Palma de Mallorca, Spain; b Hospital Son Llàtzer, Palma de Mallorca, Spain; c Institut Mediterrani d’Estudis Avançats (IMEDEA, CSIC-UIB), Universitat de les Illes Balears, Campus UIB, Palma de Mallorca, Spain; d Instituto Universitario de Investigaciones en Ciencias de la Salud (IUNICS-UIB), Universitat de les Illes Balears, Campus UIB, Palma de Mallorca, Spain

## Abstract

*Pseudomonas stutzeri* strain NF13 was isolated from a water sample taken at a hydrothermal vent in the Galapagos rift. It was selected for its ability to metabolize sulfur compounds and to grow diazotrophically. Here, we report the first draft genome of a member of genomovar 19 of the species.

## GENOME ANNOUNCEMENT

*Pseudomonas stutzeri* strain NF13 was isolated by Ruby and coworkers from a sample taken at 2,500 to 2,600 m depth in the Galapagos rift near a hydrothermal vent (1). It was assumed that H_2_S would be the predominant energy source for chemosynthesis in this habitat. Enrichment cultures under selective conditions for sulfur-oxidizing bacteria yielded several physiological groups of strains. One group, represented by strain NF13, was able to grow heterotrophically in medium containing peptone or yeast extract with or without thiosulfate. Acid was produced when thiosulfate was present. Strain NF13 grew at temperatures of 4, 22, and 35°C, but not 55°C; it was unable to utilize CO_2_ as its sole source of carbon and it was the only isolate from the samples collected that was able to fix nitrogen. It was classified as an unnamed “thiobacillus” within the *Gammaproteobacteria* (2). Recent phenotypic and phylogenetic analysis following the criteria described by Cladera et al. (3) clearly placed isolate NF13 in the *P. stutzeri* branch.

Whole-genome sequences of 6 *P. stutzeri* strains of 4 different genomovars (1, 2, 3, and 8) are publically available (4, 5, 6, 7, 8, 9). The analysis of strain NF13 will help us to gain insight into the evolution of the species by analyzing a member of a different genomovar and by studying the mechanisms of niche adaptation to deep-sea habitats by *P. stutzeri* strains.

The draft genome sequence of *P. stutzeri* strain NF13 was obtained using a total of 225,303 reads from 454 GS FLX Titanium and 500-bp Illumina HiSeq 2000 paired-end libraries. Reads were *de novo* assembled with Newbler Assembler v2.7 (Roche). The obtained genome sequence included 82 contigs (>500 bp). The calculated genome size is 4.7 Mb, and the G+C mole percent is 63.04%.

The genome prediction and annotation were performed using the NCBI Prokaryotic Genomes Automatic Annotation Pipeline (http://www.ncbi.nlm.nih.gov/genomes/static/Pipeline.html). A total of 4,321 coding sequences were identified. The frequency of individual reads is consistent with the chromosomal size and the presence of 4 copies of the genes for 5S, 16S, and 23S rRNA, as described for strains of the species (10). Genes for complete tricarboxylic acid, glycolysis, and pentose phosphate pathways are present. Furthermore, genes coding for discriminating metabolic and physiological properties of the species were detected, e.g., the complete set of genes for the denitrification pathway, for starch metabolism, and for flagellum synthesis. A complete set of nitrogen fixation genes were found. Predicted phage-related sequences, transposons, integrons, and insertion elements were detected. No extrachromosomal elements were found.

Comparative genome analysis confirmed that strain NF13 exhibited overall similarity to the 6 strains of *P. stutzeri* that have been sequenced previously (in terms of genome size and G+C content). Strain NF13 was differentiated from other genomovars by average nucleotide identity by BLAST (ANIb) values (11) ranging from 80.55 to 93.13% and it was affiliated phylogenetically with other members of *P. stutzeri* genomovar 19 also isolated from marine habitats.

### Nucleotide sequence accession numbers. 

The draft genome sequence for *P. stutzeri* NF13 has been included in the GenBank Whole-Genome Shotgun (WGS) database under the accession no. AOBS00000000. The version described is the first version, accession no. AOBS01000000.
